# Idiopathic Intracranial Hypertension Associated with SARS-CoV-2 B.1.1.7 Variant of Concern

**DOI:** 10.1017/cjn.2021.129

**Published:** 2021-06-14

**Authors:** Muhammad Faran Khalid, Jonathan A. Micieli

**Affiliations:** Michael G. DeGroote School of Medicine, McMaster University, Hamilton, Canada; Department of Ophthalmology and Vision Sciences, University of Toronto, Toronto, Canada; Division of Neurology, Department of Medicine, University of Toronto, Toronto, Canada; Kensington Vision and Research Centre, Toronto, Canada

**Keywords:** Idiopathic intracranial hypertension, *Pseudotumor cerebri*, Benign intracranial hypertension, COVID-19, SARS-CoV-2 B117

Idiopathic intracranial hypertension (IIH) is a disorder of increased intracranial pressure due to an unidentified cause.^1^ To date, cases reported of IIH associated with COVID-19 have been rare and described mainly in children. No previous cases have occurred in patients with the SARS-CoV-2 B117 variant.^2–6^ In this case study, we report on a patient diagnosed with IIH with symptoms beginning after detection of the SARS-CoV-2 B117 variant and symptoms characteristic of COVID-19.

**Figure 1:**
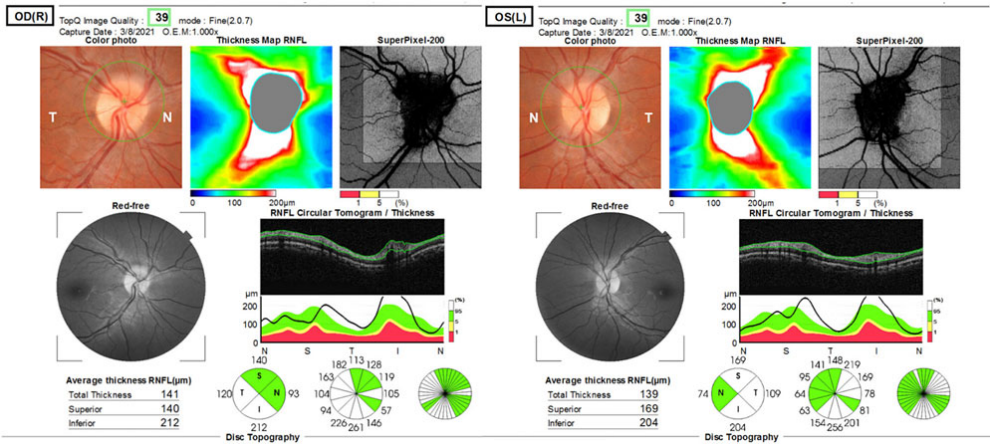
Color fundus images and optical coherence tomography of the retinal nerve fiber layer showing mild optic disk edema in the right eye with peripapillary wrinkles and mild optic disk edema in the left eye.

A 22-year-old woman (body mass index 26.5 kg/m^2^) tested positive for SARS-CoV-2 B117 variant after developing 6 days of myalgias, chills, and general malaise. She also developed new headaches a few days after onset of the aforementioned symptoms with retrobulbar pain. She did not have a history of headaches and found this unusual. The headaches were central occiput predominant, not positional and not associated with nausea or vomiting. Due to the retrobulbar pain, she went to see an optometrist 1 week after headache onset and was found to have bilateral optic disk edema (Figure 1). She was referred to the emergency department for further workup. Neuro-ophthalmic examination revealed a visual acuity of 20/20 in both eyes, no relative afferent pupillary defect and normal Humphrey 24-2 SITA-Fast visual fields. Dilated fundus examination revealed mild optic disk edema in both eyes with peripapillary wrinkles in the right eye. The mean retinal nerve fiber layer (RNFL) thickness measured by optical coherence tomography (OCT) was 141 µm right eye (OD) and 139 µm left eye (OS). Due to concern for raised intracranial pressure, she underwent magnetic resonance imaging and magnetic resonance venography of the brain, which showed flattening of the posterior globes, increased cerebrospinal fluid (CSF) space around the optic nerves, and distal transverse sinus stenosis. She underwent a lumbar puncture (LP) in left lateral decubitus position with an opening pressure of 37 cm of water and normal CSF contents. SARS-CoV-2 was not detected in the CSF. Her headaches improved after the LP. She was started on acetazolamide and her symptoms resolved after 3 weeks. At the 1-month follow-up she was asymptomatic, and the optic disk edema was essentially resolved, and OCT RNFL showed a mean thickness of 121 µm OD and 116 µm OS.

There have only been a limited number of cases reported of IIH associated with COVID-19, most of which have been in pediatric populations.^2–4^ We were able to retrieve six cases of IIH associated with COVID-19 with a mean age of 16.2 years (range of 6 years to 35 years). Only two cases were in adults, a 26-year-old female and a 35-year-old female.^5,6^ The 26-year-old presented with blurred vision and moderate papilledema, but the final visual outcome was not reported. The presence of papilledema was not reported in the 35-year-old woman who also had disorientation at presentation. Interestingly, both patients, including ours, were not obese. Our patient is the only known case of IIH in a previously healthy adult infected with the SARS-CoV-2 B117 variant that had full recovery at follow-up.

Similar to our study, in nearly all reports of increased intracranial pressure in patients with COVID-19 there has been no detection of SARS-CoV-2 RNA in the CSF, suggesting an indirect mechanism of pathogenesis.^2–6^ It is possible that the coagulation dysfunction seen in COVID-19 patients may result in venous congestion and raised intracranial pressure. Previous single-cell transcriptomics have identified de-differentiated monocytes and exhausted T cells in the CSF of patients and low-grade inflammation may also play a role.^1^ Notably, the diagnostic tool of LP has often been important in improving symptoms and providing long-term remission of IIH, as was the case for our patient. It is also possible that IIH in the context of SARS-CoV-2 is a self-limited condition.

In conclusion, IIH may be seen in the context of SARS-CoV-2 infection and should be kept in the differential diagnosis for headache and vision loss in this population. A dilated fundus examination is a reasonable next step for patient with persistent headaches after SARS-CoV-2 infection.
